# Neuron population activity in the medial prefrontal cortex suggests superimposed codes for situation and situation value

**DOI:** 10.1186/1471-2202-15-S1-P220

**Published:** 2014-07-21

**Authors:** Nathan Insel, Carol A Barnes

**Affiliations:** 1 Department of Biology, University of Toronto Scarborough, Toronto, M1C 1A4, Canada; 2 Evelyn F. McKnight Brain Institute and ARL Division of Neural Systems, Memory & Aging, University of Arizona, Tucson AZ, 85724, USA

## 

The medial prefrontal cortex (mPFC) may be necessary for an animal to use contextual information to generate appropriate behaviors[1]. Consistent with this, neuron activity in the mPFC is sensitive to an animal’s behavioral and environmental context[2,3]. The mPFC also appears to be important for determining a situation’s value: it projects strongly to other value-coding regions such as the ventral striatum, ventral tegmental area, and amygdale[4], and functional neuroimaging studies often show increased blood oxygenation in the region during high-valued situations[5]. In the present study we find evidence that the output of the mPFC includes both a high-dimensional code for an animal’s behavioral situation and, superimposed on this, a single-dimensional code for value.

Neuron ensembles and local field potentials were recorded while rats performed a novel, 3-choice, 2-cue decision task, as well as during rest epochs before and after the task. 2433 single neurons were subdivided into classes according to waveform shape and firing patterns. The majority of neurons were found to be regular-firing, putative excitatory projection neurons. Activity of individual neurons in this group carried high levels of information about behavioral context (e.g., session epoch, trial phases, space, and movement), while the population as a whole was most active near reward sites. In contrast, fast-spiking, putative inhibitory interneurons carried less information about behavioral context and fired most during rats’ acceleration or in response to task cues. The dissociations between fast-spiking and regular-firing neurons were observed even between adjacent cells with apparently reciprocal, inhibitory-excitatory connections. Notably, movement-related activity in both neuron groups was reduced in more ventral regions of the mPFC. Another, smaller population of projection neurons with burst-firing characteristics did not show clustered firing fields around rewards. This group, although heterogeneous, was also less selective for behavioral context than regular-firing cells.

Based on these results, we propose that the mPFC represents the situation an animal is in through the set of regular-firing projection neurons that are active, while also representing the value of that situation by the number of active regular firing neurons. Fast-spiking cells may normalize levels of network output according to the degree of incoming and expected sensory flow (see refs. [6,7]). Increased regular-firing neuron firing during relevant or high-value situations may result from reduced inhibition[8], or increased afferent and local network connectivity among neurons coding situation features. The presently proposed theory predicts that disruption of fast-spiking neuron function, such as is thought to occur in schizophrenia, might result in network output becoming more dependent on sensory flow and, consequently, the misattribution of value or relevance to environmental changes.
